# Error analysis for $l^{q}$-coefficient regularized moving least-square regression

**DOI:** 10.1186/s13660-018-1856-y

**Published:** 2018-09-25

**Authors:** Qin Guo, Peixin Ye

**Affiliations:** School of Mathematical Sciences and LPMC, Nankai University, Tianjin, China

**Keywords:** 68T05, 68Q32, 60E15, Moving least-square method, Data dependent hypothesis space, Regularization function, Uniform concentration inequality, Learning rate

## Abstract

We consider the moving least-square (MLS) method by the coefficient-based regression framework with $l^{q}$-regularizer $(1\leq q\leq2)$ and the sample dependent hypothesis spaces. The data dependent characteristic of the new algorithm provides flexibility and adaptivity for MLS. We carry out a rigorous error analysis by using the stepping stone technique in the error decomposition. The concentration technique with the $l^{2}$-empirical covering number is also employed in our study to improve the sample error. We derive the satisfactory learning rate that can be arbitrarily close to the best rate $O(m^{-1})$ under more natural and much simpler conditions.

## Introduction

The least-square (LS) method is an important global approximate method based on the regular or concentrated data sample points. However, there are still some irregular or scattered samples which are obtained in many practical applications such as engineering and machine learning [1–4]. They also need to be analyzed to achieve their special usefulness. For example, in geographical contour drawing, it is important to derive a set of contours but the height is available only for some scattered data sample points. Therefore, it is vital to seek a suitable local approximation method to deal with scattered data. The moving least-square (MLS) method was introduced by McLain in [4] to draw a set of contours based on a cluster of scattered data sample points. The central idea of the MLS method consists of two steps: first, one takes an arbitrary fixed point and forms a local approximation formula; second, since the fixed point is arbitrary, therefore, one can let it move over the whole domain. It turns out that MLS method is a useful local approximation tool in various mathematics fields such as approximation theory, data smoothing [5], statistics [6] and numerical analysis [7]. In computer graphics, the MLS method is useful for reconstructing a surface from a set of points. Often it is used to create a 3D surface from a point cloud. Recently, a research effort has been made to study the regression learning algorithm by the MLS method; see [8–12]. It has advantages over classical learning algorithms in the sense that its involved hypothesis space can be very simple such as the space of linear functions or a polynomial space.

We recall the regression learning problem by the MLS method briefly. Functions for learning are defined on a compact subset *X* (input space) of $\mathbb{R}^{n}$ and take values in $Y=\mathbb{R}$ (output space). The sampling process is controlled by a unknown Borel probability measure on $Z= X\times Y$. The regression function is given by

$$f_{\rho}(x)= \int_{Y} y \,d\rho(y|x), $$

where $\rho(\cdot|x)$ is the conditional probability measure induced by *ρ* on *Y* given $x\in X$. The goal of regression learning is to find a good approximation of the regression function $f_{\rho}$ based on a set of random samples $\mathbf{z}=\{z_{i}\}_{i=1}^{m}=\{(x_{i}, y_{i})\}_{i=1}^{m} \in Z^{m}$ drawn according to the measure *ρ* independently and identically.

In [11], Tong and Wu considered the following regularized MLS regression algorithm. The hypothesis space is a reproducing kernel Hilbert space (RKHS) $\mathcal{H}_{K}$ induced by a Mercer kernel *K*, which is a continuous, symmetric, and positive semi-definite function on $X\times X$. The RKHS $\mathcal {H}_{K}$ is the completion of the linear span of the set of functions $\{K_{x} :=K(x,\cdot) : x \in X \}$ with respect to the inner product $\langle\sum_{i=1}^{n} \alpha_{i} K_{x_{i}}, \sum_{j=1}^{m} \beta_{j} K_{y_{j}} \rangle_{K} := \sum_{i=1}^{n} \sum_{j=1}^{m} \alpha_{i} \beta_{j} K(x_{i} , y_{j})$. The reproducing property in $\mathcal{H}_{K}$ is

1.1 $$\begin{aligned} f(x)=\langle f, K_{x}\rangle_{K}, \quad \mbox{for all } f\in \mathcal{H}_{K}, x\in X. \end{aligned}$$

Denote $C(X)$ as the space of continuous functions on *X* with the norm $\|\cdot\|_{\infty}$. Since *K* is continuous in *X*, $\mathcal {H}_{K}\subseteq C(X)$. Let $\kappa:= \sup_{t, x\in X}|K(x,t)|<\infty $. Then, by (1.1), we have

1.2 $$\begin{aligned} \Vert f \Vert _{\infty}\leq\kappa \Vert f \Vert _{K},\quad \forall f\in\mathcal{H}_{K}, \end{aligned}$$

We define the approximation $f_{\mathbf{z},\lambda}$ of $f_{\rho}$ pointwise:

1.3 $$\begin{aligned} &f_{\mathbf{z},\lambda}(x)=f_{\mathbf{z},\sigma,\lambda ,x}(x)=f_{\mathbf{z},\sigma,\lambda,x}(u)|_{u=x}, \end{aligned}$$

1.4 $$\begin{aligned} &f_{\mathbf{z},\sigma,\lambda,x} :=\arg\min_{f\in\mathcal{H}_{K}} \Biggl\{ \frac{1}{m}\sum_{i=1}^{m}\Phi \biggl( \frac{x}{\sigma},\frac{x_{i}}{\sigma} \biggr) \bigl(y_{i}-f(x_{i}) \bigr)^{2}+\lambda \Vert f \Vert _{K}^{2} \Biggr\} , \end{aligned}$$

where $\lambda=\lambda(m)>0$ is a regularization parameter, $\sigma =\eta(m)>0$ is a window width, and $\Phi:\mathbb{R}^{n}\times \mathbb{R}^{n}\to\mathbb{R}^{+}$ is called a MLS weight function which satisfies the conditions as follows:

1.5 $$\begin{aligned} &(1)\quad 0\neq\Phi(x,t)\leq1,\quad \forall x,t \in\mathbb{R}^{n}, \end{aligned}$$

1.6 $$\begin{aligned} &(2)\quad \Phi(x,t)\geq c_{q},\quad \forall \vert x-t \vert \leq1, \end{aligned}$$

1.7 $$\begin{aligned} &(3)\quad \bigl\vert \Phi(x,t_{1})-\Phi(x,t_{2}) \bigr\vert \leq c_{\Phi} \vert t_{1}-t_{2} \vert ^{s},\quad \forall x, t_{1}, t_{2} \in \mathbb{R}^{n}, \end{aligned}$$

where the constants $q>n+1$, $c_{q}$, $c_{\Phi}>0$.

The scheme (1.3)–(1.4) shows that regularization not only ensures the computational stability but also preserves localization property for the algorithm. In this paper, we study the new regularized version of the MLS regression algorithm. We adopt the coefficient-based $l^{q}$-regularization and the data dependent hypothesis space.

1.8 $$\begin{aligned} &f_{\mathbf{z},\eta}(x)=f_{\mathbf{z},\sigma,\eta,x}(x)=f_{\mathbf {z},\sigma,\eta,x}(u)|_{u=x}, \\ \end{aligned}$$

1.9 $$\begin{aligned} &f_{\mathbf{z},\sigma,\eta,x}=\arg\min_{f\in\mathcal{H}_{K, \mathbf{z}} } \Biggl\{ \frac{1}{m}\sum_{i=1}^{m}\Phi \biggl( \frac{x}{\sigma},\frac {x_{i}}{\sigma} \biggr) \bigl(f(x_{i})-y_{i} \bigr)^{2}+\eta\Omega_{\mathbf{z}}(f) \Biggr\} ,\quad \eta=\eta(m)>0, \end{aligned}$$

where

$$\begin{aligned} &\mathcal{H}_{K, \mathbf{z}}= \Biggl\{ f(x)=\sum_{i=1}^{m} \alpha_{i}K(x,x_{i}):\alpha =(\alpha_{1},\ldots, \alpha_{m})\in\mathbb{R}^{m},m\in \mathbb{N} \Biggr\} , \\ &\Omega_{\mathbf{z}}(f)=\inf \Biggl\{ \sum_{i=1}^{m} \vert \alpha _{i} \vert ^{q} \Biggr\} ,\quad 1\leq q\leq2. \end{aligned}$$

The data dependence nature of the kernel-based hypothesis space provides flexibility for the learning algorithm such as choosing the $l^{q}$-norm regularizer of a function expansion involving samples. Compared with the scheme (1.3)–(1.4) in a reproducing kernel Hilbert space, the first advantage of the algorithm (1.9) is the effectivity of computations without any optimization processes. Another advantage is that we can choose the suitable parameter *q* according to the research interest such as smoothness and sparsity. To study the approximation quality of $f_{\mathbf{z},\eta}$, we derive the upper bound of the error $\| f_{\mathbf{z},\eta}-f_{\rho}\|_{\rho_{X}}$ with $\|f(\cdot)\|_{\rho_{X}}:=(\int_{X}|f(\cdot)|^{2}d{\rho _{X}})^{\frac{1}{2}}$ and its convergence rates as $m \to\infty$; see [8–11, 13, 14]. The remainder of this paper is organized as follows. In Sect. 2, we will provide the main result. The error decomposition analysis and the upper bounds of the hypothesis error, the approximation error and the sample error will be given in Sects. 3. In Sect. 4, we will prove the main result. Finally, Sect. 5 concludes the paper with future research lines.

## Main result

We firstly formulate some basic notations and assumptions.

Let $\rho_{X}$ be the marginal distribution of *ρ* on *X* and $L_{\rho_{X}} ^{2}(X)$ be the Hilbert space of functions from *X* to *Y* square-integrable with respect to $\rho_{X}$ with the norm denoted by $\|\cdot\|_{\rho _{X}}$. The integral operator $L_{K}:L_{\rho_{X}} ^{2}(X)\rightarrow L_{\rho _{X}} ^{2}(X)$ is defined by

$$(L_{K} f) (x) = \int_{X} K(x,t)f(t)\,d\rho_{X}(t),\quad x\in X. $$

Since *X* is compact and *K* is continuous, $L_{K}$ is a compact operator. Its fractional power operator $L_{K}^{r}:L_{\rho_{X}} ^{2}(X)\rightarrow L_{\rho_{X}} ^{2}(X), r>0$ is defined by

$$L_{K}^{r}(f)=\sum_{i=1}^{\infty} \mu_{i}^{r}\langle f,e_{i}\rangle _{L_{\rho_{X}} ^{2}}e_{i},\quad f\in L_{\rho_{X}} ^{2}(X), $$

where $\{\mu_{i}\} $ are the eigenvalues of the operator $L_{K}$ and $\{e_{i}\}$ are the corresponding eigenfunctions which form an orthonormal basis of $L_{\rho_{X}} ^{2}(X)$; see [15]. For $r>0$, the function $f_{\rho}$ is said to satisfy the regularity condition of order *r* provided that $L_{K}^{-r}f_{\rho}\in L^{2}_{\rho_{X}}$.

We show the following nice feature for the capacity of $\mathcal {H}_{K, \mathbf{z}}$ when the $l^{2}$-empirical covering number is used; see [16],

2.1 $$\begin{aligned} \log\mathcal {N}_{2}(B_{1},\epsilon)\leq c_{p}\epsilon^{-p},\quad \forall\epsilon>0, \end{aligned}$$

where $B_{1}= \{f\in\mathcal{H}_{K, \mathbf{z}}: \|f\|_{K}\leq 1 \}$, the exponent $0< p<2$ and the constant $c_{p}>0$.

### Definition 2.1

The probability measure $\rho_{X} $ on *X* is said to satisfy the condition $L_{\tau}$ with exponent $\tau>0$ if

2.2 $$\begin{aligned} \rho_{X} \bigl(B(x,r)\bigr)\geq c_{\tau}r^{\tau},\quad \forall 0< r\leq r_{0}, x\in X, \end{aligned}$$

where the constants $r_{0}>0$, $c_{\tau}>0$ and $B(x,r)=\{u\in X: |u-x|\leq r\}$ for $r>0$.

We use the projection operator to obtain the faster learning rate under the condition $|y|\leq M$ and $M\geq1$ almost surely; see [17–19].

### Definition 2.2

Fix $M>0$, the projection operator $\pi_{M}$ on the space of measurable functions $f:X\rightarrow\mathbb{R}$ is defined as

2.3 $$\begin{aligned} \pi_{M}(f) (x)= \textstyle\begin{cases}M, & \mbox{if } f(x)>M, \\f(x), & \mbox{if } \vert f(x) \vert \leq M, \\ -M, & \mbox{if } f(x)< -M. \end{cases}\displaystyle \end{aligned}$$

We assume all the constants are positive and independent of *δ*, *m*, *λ*, *η* or *σ*. Now we are in a position to give the learning rates of the algorithm (1.9).

### Theorem 2.1

*Suppose* $L_{K}^{-r}f_{\rho}\in L^{2}_{\rho_{X}}$ *with* $r>0$, (2.1) *with* $0< p<2$ *and* (2.2) *hold*. *If all the functions* $f\in\mathcal{H}_{K}\cup\{{f_{\rho}}\}$ *satisfy the Lipschitz condition on* *X*, *that is*, *for the constant* $c_{0}>0$,

2.4 $$\begin{aligned} \bigl\vert f(u)-f(v) \bigr\vert \leq c_{0} \vert u-v \vert , \quad \forall u, v\in X, \end{aligned}$$

*then*, *for any* $0<\delta<1$, *with confidence* $1-\delta$, *we have*

2.5 $$ \bigl\Vert \pi_{M}(f_{\mathbf{z},\eta}) - f_{\rho}\bigr\Vert _{\rho_{X}}^{2} \leq \widetilde{D} \biggl(\frac{1}{m} \biggr)^{\theta(r)}\log \biggl(\frac {2}{\delta} \biggr), $$

*where*

$$\theta(r) = \textstyle\begin{cases}\min \{\frac{q}{[r(2p+2q+pq)+pq]}, 1 \} (\frac{2r}{1+\tau} ), & 0< r< \frac{1}{2}; \\ \frac{2q}{(2p+2q+3pq)(1+\tau)}, & r\geq1/2. \end{cases} $$

### Remark 2.1

When $p\rightarrow0$ and $r\geq\frac{1}{2}$, our convergence rate $m^{-\frac{2q}{(2p+2q+3pq)(1+\tau)}}$ tends to $m^{-\frac{1}{1+\tau }}$. In [11], the authors have derived the rate $m^{-\frac{1}{1+\tau}}$. In particular, assuming the unnatural norm condition in [8] holds, we can obtain the faster rate $m^{\tau\varepsilon-\frac{2q}{2p+2q+3pq}}$ for $r\geq\frac {1}{2}$, which can be arbitrarily close to $O(m^{-1})$ as $\varepsilon \rightarrow0$ and $p\rightarrow0$.

## Error analysis

We only present the results of the main propositions in this section. All the proofs will be given in the appendix. To estimate $\|\pi_{M}(f_{\mathbf{z},\eta})-f_{\rho}\|^{2}_{\rho_{X}}$, we invoke the following proposition, whose proof is completely similar to that of Theorem 3.3 in [11].

### Proposition 3.1

*If* $\rho_{X}$ *satisfies* (2.1), *and all the functions* $f\in \mathcal{H}_{K}\cup\{{f_{\rho}}\}$ *satisfy* (2.4), *then*

3.1 $$\begin{aligned} \bigl\Vert \pi_{M}(f_{\mathbf{z},\eta})-f_{\rho } \bigr\Vert ^{2}_{\rho_{X}}\leq\frac{\sigma^{-\tau}}{c_{q}c_{\tau}} \int_{X} \bigl\{ \mathcal{E}_{x}\bigl( \pi_{M}(f_{\mathbf{z},\sigma,\eta ,x})\bigr)-\mathcal{E}_{x}(f_{\rho}) \bigr\} \,d\rho_{X}(x)+8c_{0}M\sigma, \end{aligned}$$

*where*

3.2 $$\begin{aligned} \mathcal{E}_{x}(f)= \int_{Z}\Phi \biggl(\frac{x}{\sigma},\frac {u}{\sigma} \biggr) \bigl(f(u)-y\bigr)^{2}\,d\rho(u,y), \quad \forall f: X\rightarrow \mathbb{R} \end{aligned}$$

*is called the local moving expected risk*.

Then we only need to provide the upper bound of the integral in (3.1). So to do this, we give its decomposition by using $f_{\mathbf{z},\lambda}$, which plays a stepping stone role between $f_{\mathbf{z},\eta}$ and the regularization function $f_{\lambda}$, while different regularization parameters *λ* and *η* are adopted. Here $f_{\lambda}$ is given by

3.3 $$\begin{aligned} f_{\lambda}:=\arg\min_{f\in\mathcal{H}_{K}} \bigl\{ \Vert f-f_{\rho} \Vert _{\rho_{X}}^{2}+\lambda \Vert f \Vert _{K}^{2} \bigr\} . \end{aligned}$$

### Proposition 3.2

*Let* $f_{\mathbf{z},\sigma,\eta,x}$ *be defined as in* (1.9) *and*

3.4 $$\begin{aligned} \mathcal{E}_{\mathbf{z},x}(f)=\frac{1}{m}\sum _{i=1}^{m}\Phi \biggl(\frac{x}{\sigma}, \frac{x_{i}}{\sigma} \biggr) \bigl(f(x_{i})-y_{i} \bigr)^{2} \end{aligned}$$

*be the local moving empirical risk*. *Then*

3.5 $$\begin{aligned} \int_{X} \bigl\{ \mathcal{E}_{x}\bigl( \pi_{M}(f_{\mathbf{z},\sigma,\eta ,x})\bigr)-\mathcal{E}_{x}(f_{\rho}) \bigr\} \,d\rho_{X}(x)\leq\mathcal {S}(\mathbf{z},\lambda,\eta)+ \mathcal{H}(\mathbf{z},\lambda,\eta )+\mathcal{D}(\lambda), \end{aligned}$$

*where*

$$\begin{aligned} &\begin{aligned} \mathcal{S}(\mathbf{z},\lambda,\eta)&= \int_{X} \bigl\{ \mathcal {E}_{x}\bigl( \pi_{M}(f_{\mathbf{z}, \sigma,\eta,x})\bigr)-\mathcal {E}_{\mathbf{z},x}\bigl( \pi_{M}(f_{\mathbf{z}, \sigma,\eta ,x})\bigr) \\ &\quad {}+\mathcal{E}_{\mathbf {z},x}(f_{\lambda})- \mathcal{E}_{x}(f_{\lambda}) \bigr\} \,d\rho _{X}(x), \end{aligned} \\ &\begin{aligned}\mathcal{H}(\mathbf{z},\lambda,\eta)&= \int_{X} \bigl\{ \bigl(\mathcal {E}_{\mathbf{z},x}\bigl( \pi_{M}(f_{\mathbf{z}, \sigma,\eta,x})\bigr)+\eta \Omega_{\mathbf{z}}(f_{\mathbf{z}, \sigma,\eta,x}) \bigr) \\ &\quad {}-\bigl(\mathcal{E}_{\mathbf{z},x}(f_{\lambda })+ \lambda \Vert f_{\lambda} \Vert _{K}^{2}\bigr) \bigr\} \,d\rho_{X}(x), \end{aligned} \\ &\mathcal{D}(\lambda)= \Vert f_{\lambda}-f_{\rho} \Vert _{\rho _{X}}^{2}+\lambda \Vert f_{\lambda} \Vert _{K}^{2}. \end{aligned}$$

$\mathcal{S}(\mathbf{z},\lambda,\eta)$ is known as the sample error. $\mathcal{H}(\mathbf{z},\lambda,\eta)$ is called the hypothesis error. $\mathcal{D}(\lambda)$ is called the approximation error.

The estimation of the hypothesis error can be conducted analogously to that in [18].

### Proposition 3.3

*Under the assumptions of Theorem* 2.1, *we have*

$$\begin{aligned} \mathcal{H}(\mathbf{z},\lambda,\eta)\leq\frac{m\eta M^{2}}{(m\lambda)^{q}}. \end{aligned}$$

For the approximation error, we directly invoke the following result in [20].

### Proposition 3.4

*Under the assumption* $L_{K}^{-r}f_{\rho}\in L^{2}_{\rho_{X}}$ *with* $r>0$, *we have*

3.6 $$\begin{aligned} \mathcal{D}(\lambda)\leq C_{1}\lambda^{\min\{2r,1\}}. \end{aligned}$$

For the sample error, we decompose it into two parts:

$$\begin{aligned} \mathcal{S}(\mathbf{z},\lambda,\eta)&= \int_{X} \bigl\{ \mathcal {E}_{x}\bigl( \pi_{M}(f_{\mathbf{z}, \sigma,\eta,x})\bigr)-\mathcal {E}_{x}(f_{\rho}) \\ &\quad{}-\mathcal{E}_{\mathbf{z},x}\bigl(\pi _{M}(f_{\mathbf{z}, \sigma,\eta,x}) \bigr)+\mathcal{E}_{\mathbf {z},x}(f_{\rho}) \bigr\} \,d\rho_{X}(x) \\ &\quad{}+ \int_{X} \bigl\{ \mathcal{E}_{\mathbf{z},x}(f_{\lambda })- \mathcal{E}_{\mathbf{z},x}(f_{\rho}) \\ &\quad{}-\mathcal {E}_{x}(f_{\lambda})+\mathcal{E}_{x}(f_{\rho}) \bigr\} \,d\rho _{X}(x) \\ &:=\mathcal{S}_{1}(\mathbf{z},\eta)+\mathcal{S}_{2}(\mathbf {z},\lambda). \end{aligned}$$

We firstly give the upper bound of $\mathcal{S}_{2}(\mathbf {z},\lambda)$ by using the Bernstein probability inequality in [14, 21].

### Proposition 3.5

*Under the assumptions of Theorem* 2.1, *for any* $0<\delta <1$, *with confidence* $1-\delta/2$,

3.7 $$\begin{aligned} \mathcal{S}_{2}(\mathbf{z},\lambda)\leq \frac{\mathcal{D}(\lambda)}{2}+\frac{7 (3M+\kappa\sqrt{\frac {\mathcal{D}(\lambda)}{\lambda}} )^{2}\log(2/\delta)}{3m}. \end{aligned}$$

Next the estimation for $\mathcal{S}_{1}(\mathbf{z},\eta)$ is more difficult in the sense that it involves the complexity of the function space $\mathcal{H}_{K, \mathbf{z}}$. Hence we need the uniform concentration inequality from [22].

### Proposition 3.6

*Under the assumptions of Theorem* 2.1, *for any* $0<\delta <1$, *with confidence* $1-\delta/2$,

3.8 $$\begin{aligned} \mathcal{S}_{1}(\mathbf{z},\eta)&\leq\frac{1}{2} \int_{X} \bigl\{ \mathcal{E}_{x}\bigl( \pi_{M}(f_{\mathbf{z}, \sigma,\eta,x})\bigr)-\mathcal {E}_{x}(f_{\rho}) \bigr\} \,d\rho_{X}(x) \\ &\quad {}+\frac{176M^{2}}{m}\log \biggl(\frac{2}{\delta} \biggr)+C_{p,M}R_{\eta}^{\frac{2p}{2+p}}m^{-\frac{2}{2+p}}, \end{aligned}$$

*where* $R_{\eta}=\kappa m^{1-\frac{1}{q}} (\frac{M^{2}}{\eta } )^{\frac{1}{q}}$.

## Proof of the main result

Now we derive the learning rates.

### Proof of Theorem 2.1

Combining the four bounds of Proposition 3.3, 3.4, 3.5 and 3.6 with (3.5), with confidence $1-\delta$, we have

4.1 $$\begin{aligned} & \int_{X} \bigl\{ \mathcal{E}_{x}\bigl( \pi_{M}(f_{\mathbf{z},\sigma,\eta ,x})\bigr)-\mathcal{E}_{x}(f_{\rho}) \bigr\} \,d\rho_{X}(x) \\ &\quad \leq D_{1}\log \biggl(\frac{2}{\delta} \biggr) \bigl\{ \lambda^{\min \{2r,1\}}+ m^{-1}\lambda^{\min\{2r-1,0\}} \\ &\qquad{}+m^{1-q}\eta\lambda^{-q}+m^{\frac{-2q-2p+2pq}{(2+p)q}}\eta ^{-\frac{2p}{q(2+p)}} \bigr\} . \end{aligned}$$

By substituting (4.1) into (3.1), we have

$$\begin{aligned} \bigl\Vert \pi_{M}(f_{\mathbf{z},\eta})-f_{\rho} \bigr\Vert ^{2}_{\rho _{X}}&\leq D_{2}\log \biggl(\frac{2}{\delta} \biggr) \bigl\{ \sigma ^{-\tau} \bigl\{ \lambda^{\min\{2r,1\}}+ m^{-1}\lambda^{\min\{ 2r-1,0\}} \\ &\quad{}+m^{1-q}\eta\lambda^{-q}+m^{\frac{-2q-2p+2pq}{(2+p)q}}\eta ^{-\frac{2p}{q(2+p)}} \bigr\} +\sigma \bigr\} . \end{aligned}$$

When $0< r<1/2$,

$$\begin{aligned} \bigl\Vert \pi_{M}(f_{\mathbf{z},\eta})-f_{\rho} \bigr\Vert ^{2}_{\rho _{X}}&\leq D_{2}\log \biggl(\frac{2}{\delta} \biggr) \bigl\{ \sigma ^{-\tau} \bigl\{ \lambda^{2r}+ m^{-1}\lambda^{2r-1}+m^{1-q}\eta \lambda^{-q} \\ &\quad{}+m^{\frac{-2q-2p+2pq}{(2+p)q}}\eta^{-\frac{2p}{q(2+p)}} \bigr\} +\sigma \bigr\} . \end{aligned}$$

Let $\lambda=m^{-\theta_{1}}$, $\eta=m^{-\theta_{2}}$ and $\sigma =m^{-\theta_{3}}$.

4.2 $$\begin{aligned} \bigl\Vert \pi_{M}(f_{\mathbf{z},\eta})-f_{\rho } \bigr\Vert ^{2}_{\rho_{X}}\leq D_{3}\log \biggl( \frac{2}{\delta} \biggr) m^{-\theta}, \end{aligned}$$

where

$$\begin{aligned} \theta&=\min \biggl\{ -\tau\theta_{3}+2r\theta_{1}, -\tau \theta_{3}+1+(2r-1)\theta_{1}, \\ &\quad{}-\tau\theta_{3}+q-1+\theta_{2}-q \theta_{1}, \\ &\quad{}-\tau\theta_{3}+\frac{2q+2p-2pq}{(2+p)q}-\frac {2p}{q(2+p)} \theta_{2}, \theta_{3} \biggr\} . \end{aligned}$$

To maximize the learning rate, we take

$$\begin{aligned} \theta_{\max}&=\max_{\theta_{1}, \theta_{3}}\min \biggl\{ \max _{\theta_{2}}\min \biggl\{ -\tau\theta_{3}+q-1+\theta _{2}-q\theta_{1}, \\ &\quad {} -\tau\theta_{3}+\frac {2q+2p-2pq}{(2+p)q}- \frac{2p}{q(2+p)}\theta_{2} \biggr\} , \\ &\quad {} -\tau\theta_{3}+2r\theta_{1}, -\tau \theta _{3}+1+(2r-1)\theta_{1}, \theta_{3} \biggr\} . \end{aligned}$$

Let

$$\begin{aligned} -\tau\theta_{3}+q-1+\theta_{2}-q\theta_{1}&=- \tau\theta_{3}+\frac {2q+2p-2pq}{(2+p)q}-\frac{2p}{q(2+p)} \theta_{2}. \end{aligned}$$

Then

$$\begin{aligned} \theta_{\max}&=\max_{\theta_{1}, \theta_{3}}\min \biggl\{ -\tau \theta_{3}+q-1-q\theta_{1} +\frac {-pq+4q+2p-2q^{2}-pq^{2}}{2p+2q+pq} \\ &\quad{} +\frac{(2+p)q^{2}}{2p+2q+pq}\theta_{1},-\tau \theta_{3}+2r\theta_{1}, \\ &\quad{} -\tau\theta_{3}+1+(2r-1)\theta _{1}, \theta_{3} \biggr\} \\ &\geq\max_{\theta_{3}}\min \biggl\{ \max_{\theta_{1}}\min \biggl\{ -\tau\theta_{3}+q-1-q\theta_{1} \\ &\quad{} +\frac {-pq+4q+2p-2q^{2}-pq^{2}}{2p+2q+pq} \\ &\quad{} +\frac{(2+p)q^{2}}{2p+2q+pq}\theta_{1},-\tau \theta_{3}+2r\theta_{1} \biggr\} , \\ &\quad {} \max_{\theta_{1}}\min \bigl\{ -\tau \theta_{3}+1+(2r-1)\theta_{1},-\tau\theta_{3}+2r \theta _{1} \bigr\} , \theta_{3} \biggr\} . \end{aligned}$$

Let

$$\begin{aligned} &{-}\tau\theta_{3}+q-1-q\theta_{1}+\frac {-pq+4q+2p-2q^{2}-pq^{2}}{2p+2q+pq} \\ &\quad {}+\frac{(2+p)q^{2}}{2p+2q+pq}\theta_{1}=-\tau\theta_{3}+2r\theta _{1}, \\ &{-}\tau\theta_{3}+1+(2r-1)\theta_{1}=-\tau \theta_{3}+2r\theta _{1}. \end{aligned}$$

Then

$$\begin{aligned} \theta_{\max}&\geq\max_{\theta_{3}}\min \biggl\{ -\tau \theta_{3}+\frac{4qr}{2r(2p+2q+pq)+2pq},-\tau\theta_{3}+2r,\theta _{3} \biggr\} \\ &\geq\min \biggl\{ \max_{\theta_{3}}\min \biggl\{ -\tau\theta _{3}+\frac{4qr}{2r(2p+2q+pq)+2pq},\theta_{3} \biggr\} , \\ &\quad {} \max_{\theta_{3}}\min \{-\tau \theta_{3}+2r, \theta_{3} \} \biggr\} \\ &=2r\min \biggl\{ -\frac{q\tau}{(1+\tau)[r(2p+2q+pq)+pq]} \\ &\quad {}+\frac{q}{r(2p+2q+pq)+pq}, \frac {-\tau}{1+\tau}+1 \biggr\} . \end{aligned}$$

When $r\geq1/2$,

$$\begin{aligned} \bigl\Vert \pi_{M}(f_{\mathbf{z},\eta})-f_{\rho} \bigr\Vert ^{2}_{\rho _{X}}&\leq D_{2}\log \biggl(\frac{2}{\delta} \biggr) \bigl\{ \sigma ^{-\tau} \bigl\{ \lambda+ m^{-1}+m^{1-q} \eta\lambda^{-q} \\ &\quad{}+m^{\frac{-2q-2p+2pq}{(2+p)q}}\eta^{-\frac{2p}{q(2+p)}} \bigr\} +\sigma \bigr\} . \end{aligned}$$

Similarly, we obtain

$$\begin{aligned} \theta_{\max}\geq\frac{2q}{(1+\tau )(2p+2q+3pq)}. \end{aligned}$$

So we choose

$$\theta(r) = \textstyle\begin{cases}\min \{\frac{q}{[r(2p+2q+pq)+pq]}, 1 \} (\frac{2r}{1+\tau} ), & 0< r< \frac{1}{2}; \\ \frac{2q}{(2p+2q+3pq)(1+\tau)}, & r\geq1/2. \end{cases} $$

We complete the proof of Theorem 2.1. □

## Conclusion and further discussion

We obtain the upper error bound of the algorithm (1.9) for the independent and identical samples with $1\leq q \leq2$. We decomposed the error quantity into the approximation error, the hypothesis error and the sample error and obtained their upper bounds using error analysis techniques developed in learning theory. In some practical applications, we may often encounter the non-i.i.d. sampling processes such as weakly dependent or non-identical processes; see [13, 15, 20]. It may be interesting to continue our error analysis for the non-i.i.d. samples.

## Appendix 1: Error decomposition

### Proof of Proposition 3.2

We have

A.1 $$\begin{aligned} & \int_{X} \bigl\{ \mathcal{E}_{x}\bigl( \pi_{M}(f_{\mathbf{z},\sigma ,\eta,x})\bigr)-\mathcal{E}_{x}(f_{\rho}) \bigr\} \,d\rho_{X}(x) \\ &\quad \leq \int_{X} \bigl\{ \mathcal{E}_{x}\bigl( \pi_{M}(f_{\mathbf{z},\sigma ,\eta,x})\bigr)-\mathcal{E}_{x}(f_{\rho})+ \eta\Omega_{\mathbf{z}}(f_{\mathbf{z}, \sigma,\eta,x}) \bigr\} \,d\rho_{X}(x) \\ &\quad =\mathcal{S}(\mathbf{z},\lambda,\eta)+\mathcal{H}(\mathbf {z},\lambda,\eta)+ \int_{X} \bigl\{ \mathcal{E}_{x}(f_{\lambda })- \mathcal{E}_{x}(f_{\rho})+\lambda \Vert f_{\lambda} \Vert _{K}^{2} \bigr\} \,d\rho_{X}(x). \end{aligned}$$

Moreover, by (1.5),

A.2 $$\begin{aligned} \mathcal{E}_{x}(f_{\lambda})- \mathcal{E}_{x}(f_{\rho})&= \int _{X}\Phi \biggl(\frac{x}{\sigma},\frac{u}{\sigma} \biggr) \bigl(f_{\lambda}(u)-f_{\rho}(u)\bigr)^{2}\,d \rho_{X}(u) \\ &\leq \Vert f_{\lambda}-f_{\rho} \Vert _{\rho_{X}}^{2}. \end{aligned}$$

This completes the proof of Proposition 3.2. □

## Appendix 2: Estimates for the hypothesis error

### Proof of Proposition 3.3

It is known from Theorem 2.1 in [11] that the coefficient ${\mathbf{a}}^{\mathbf{z}}=(a_{1}^{\mathbf{z}},\ldots ,a_{m}^{\mathbf{z}})^{T}$ of $f_{\mathbf{z}, \sigma,\lambda,x}$ satisfies

$$\begin{aligned} \bigl(m\lambda I+Q_{x}K[{\mathbf{x}}]\bigr){\mathbf{a}}^{\mathbf {z}}=Q_{x}{ \mathbf{y}}, \end{aligned}$$

where *I* is the identity matrix, ${\mathbf{y}}=(y_{1}, y_{2}, y_{3},\ldots, y_{m})^{T}$, $K[{\mathbf {x}}]=(K(x_{i},x_{j}))_{i,j=1}^{m}$ and $Q_{x}=\operatorname{diag} (\Phi (\frac{x}{\sigma},\frac{x_{i}}{\sigma} ):i=1,2,\ldots,m )$.

This implies

$$\begin{aligned} \lambda m{\mathbf{a}}^{\mathbf{z}}=Q_{x}\bigl({\mathbf {y}}-K[{ \mathbf{x}}]{\mathbf{a}}^{\mathbf{z}}\bigr). \end{aligned}$$

Therefore, for $i=1,2,\ldots,m$, we get

$$\begin{aligned} a_{i}^{\mathbf{z}}&=\frac{1}{\lambda m}\Phi \biggl( \frac {x}{\sigma},\frac{x_{i}}{\sigma} \biggr) \Biggl(y_{i}-\sum_{j=1}^{m}a_{j}^{\mathbf{z}}K(x_{i},x_{j}) \Biggr) \\ &=\frac {1}{\lambda m}\Phi \biggl(\frac{x}{\sigma},\frac{x_{i}}{\sigma} \biggr) \bigl(y_{i}-f_{\mathbf{z}, \sigma,\lambda,x}(x_{i})\bigr). \end{aligned}$$

By the Hölder inequality, we have

$$\begin{aligned} \sum_{i=1}^{m} \bigl\vert a_{i}^{\mathbf{z}} \bigr\vert ^{q}&=\frac {1}{(\lambda m)^{q}} \sum_{i=1}^{m} \biggl\vert \Phi \biggl( \frac{x}{\sigma },\frac{x_{i}}{\sigma} \biggr) \bigl(y_{i}-f_{\mathbf{z}, \sigma,\lambda ,x}(x_{i}) \bigr) \biggr\vert ^{q} \\ &\leq\frac{1}{(\lambda m)^{q}} \Biggl(\sum_{i=1}^{m} \Phi \biggl(\frac{x}{\sigma},\frac{x_{i}}{\sigma } \biggr)^{\frac{q}{2-q}} \Biggr)^{1-\frac{q}{2}} \\ &\quad\times{} \Biggl(\sum_{i=1}^{m} \biggl( \frac{x}{\sigma},\frac {x_{i}}{\sigma} \biggr) \bigl(y_{i}-f_{\mathbf{z}, \sigma,\lambda ,x}(x_{i}) \bigr)^{2} \Biggr)^{\frac{q}{2}}. \end{aligned}$$

By (1.5), we have

$$\begin{aligned} \sum_{i=1}^{m} \bigl\vert a_{i}^{\mathbf{z}} \bigr\vert ^{q}\leq \frac{m}{(\lambda m)^{q}} \bigl(\mathcal{E}_{\mathbf{z},x}(f_{\mathbf{z}, \sigma ,\lambda,x}) \bigr)^{\frac{q}{2}}. \end{aligned}$$

Thus,

$$\begin{aligned} &\mathcal{E}_{\mathbf{z},x}\bigl(\pi_{M}(f_{\mathbf{z}, \sigma ,\eta,x}) \bigr)+\eta\Omega_{\mathbf{z}}(f_{\mathbf{z}, \sigma,\eta ,x}) \\ &\quad \leq\mathcal{E}_{\mathbf{z},x}(f_{\mathbf{z}, \sigma,\eta,x})+\eta\Omega_{\mathbf{z}}(f_{\mathbf{z}, \sigma ,\eta,x}) \\ &\quad \leq\mathcal{E}_{\mathbf{z},x}(f_{\mathbf {z}, \sigma,\lambda,x})+\eta\Omega_{\mathbf{z}}(f_{\mathbf{z}, \sigma,\lambda,x}) \\ &\quad \leq\mathcal{E}_{\mathbf {z},x}(f_{\mathbf{z}, \sigma,\lambda,x})+\frac{m\eta}{(\lambda m)^{q}} \bigl( \mathcal{E}_{\mathbf{z},x}(f_{\mathbf{z}, \sigma ,\lambda,x}) \bigr)^{\frac{q}{2}} \\ &\quad \leq\mathcal {E}_{\mathbf{z},x}(f_{\mathbf{z}, \sigma,\lambda,x})+\lambda \Vert f_{\mathbf{z}, \sigma,\lambda,x} \Vert _{K}^{2} \\ &\qquad{}+\frac {m\eta}{(\lambda m)^{q}} \bigl(\mathcal{E}_{\mathbf{z},x}(f_{\mathbf {z}, \sigma,\lambda,x})+ \lambda \Vert f_{\mathbf{z}, \sigma,\lambda ,x} \Vert _{K}^{2} \bigr)^{\frac{q}{2}}. \end{aligned}$$

Since

$$\begin{aligned} \mathcal{E}_{\mathbf{z},x}(f_{\mathbf{z}, \sigma,\lambda ,x})+\lambda \Vert f_{\mathbf{z}, \sigma,\lambda,x} \Vert _{K}^{2}\leq \mathcal{E}_{\mathbf{z},x}(0)+\lambda \Vert 0 \Vert _{K}^{2}, \end{aligned}$$

we get

B.1 $$\begin{aligned} &\mathcal{E}_{\mathbf{z},x}\bigl(\pi_{M}(f_{\mathbf{z}, \sigma ,\eta,x}) \bigr)+\eta\Omega_{\mathbf{z}}(f_{\mathbf{z}, \sigma,\eta ,x}) \\ &\quad\leq\mathcal{E}_{\mathbf{z},x}(f_{\mathbf{z}, \sigma,\lambda,x})+\lambda \Vert f_{\mathbf{z}, \sigma,\lambda,x} \Vert _{K}^{2}+\frac{m\eta M^{2}}{(\lambda m)^{q}}. \end{aligned}$$

It follows from (1.3)–(1.4) that

B.2 $$\begin{aligned} \mathcal{E}_{\mathbf{z},x}(f_{\mathbf{z}, \sigma,\lambda ,x})+\lambda \Vert f_{\mathbf{z}, \sigma,\lambda,x} \Vert _{K}^{2}\leq \mathcal{E}_{\mathbf{z},x}(f_{\lambda})+ \lambda \Vert f_{\lambda} \Vert _{K}^{2}. \end{aligned}$$

Combining (B.1) and (B.2), we obtain our desired estimation. □

## Appendix 3: Estimates for the sample error

We estimate $\mathcal{S}_{2}(\mathbf{z},\lambda)$ by using the lemma in [14, 21] below.

### Lemma C.1

*Let* $\{\xi(z_{i})\}_{i=1}^{m}$ *be independent random variables on a probability space* *Z* *with mean* $\mathbb{E}(\xi)$ *and variance* $\sigma^{2}(\xi)$. *Assume* $|\xi({z})-\mathbb{E}\xi|\leq M$ *almost surely*. *Then*, *for any* $0<\delta<1$, *with confidence* $1-\delta$, *we have*

$$\begin{aligned} \frac{1}{m}\sum_{i=1}^{m} \xi(z_{i})-\mathbb{E}\xi\leq\frac {2M\log(1/\delta)}{3m}+ \sqrt{ \frac{2\sigma^{2}(\xi)\log (1/\delta)}{m}}. \end{aligned}$$

### Proof of Proposition 3.5

Let $g(u,y)=\int_{X}\Phi (\frac{x}{\sigma},\frac{u}{\sigma } )[(y-f_{\lambda}(u))^{2}-(y-f_{\rho}(u))^{2}]\,d\rho_{X}(x)$, for any $z=(u,y)\in Z$. Then

$$\begin{aligned} & \int_{Z}g\,d\rho= \int_{X} \bigl\{ \mathcal{E}_{x}(f_{\lambda })- \mathcal{E}_{x}(f_{\rho}) \bigr\} \,d\rho_{X}(x); \\ &\frac{1}{m}\sum_{i=1}^{m}g(z_{i})= \int_{X} \bigl\{ \mathcal {E}_{\mathbf{z},x}(f_{\lambda})- \mathcal{E}_{\mathbf{z},x}(f_{\rho }) \bigr\} \,d\rho_{X}(x). \end{aligned}$$

By (1.2) and (3.3), we have

$$\begin{aligned} \Vert f_{\lambda} \Vert _{\infty}\leq\kappa \Vert f_{\lambda} \Vert _{K}\leq\kappa \sqrt{\frac{\mathcal{D}(\lambda)}{\lambda}}. \end{aligned}$$

Combining with (1.5), we have

$$\begin{aligned} \bigl\vert g(u,y) \bigr\vert &\leq \int_{X}\Phi \biggl(\frac{x}{\sigma},\frac{u}{\sigma } \biggr) \bigl\vert \bigl(f_{\lambda}(u)-f_{\rho}(u) \bigr) \\ &\quad{}\times \bigl(f_{\lambda}(u)+f_{\rho}(u)-2y \bigr) \bigr\vert \,d\rho _{X}(x) \\ &\leq\bigl( \Vert f_{\lambda} \Vert _{\infty}+M\bigr) \bigl(3M+ \Vert f_{\lambda} \Vert _{\infty }\bigr) \\ &\leq \biggl(3M+\kappa\sqrt{\frac{\mathcal{D}(\lambda)}{\lambda }} \biggr)^{2}:=B_{\lambda}. \end{aligned}$$

Therefore,

$$\biggl\Vert g(u,y)- \int_{Z}g\,d\rho \biggr\Vert _{\infty} \leq2B_{\lambda} $$

and

$$\begin{aligned} \sigma^{2}(g)&\leq \int_{Z}g^{2}\,d\rho \\ &= \int_{Z} \biggl( \int_{X}\Phi \biggl(\frac{x}{\sigma},\frac {u}{\sigma} \biggr)\,d\rho_{X}(x) \biggr)^{2} \bigl(f_{\lambda}(u)-f_{\rho }(u) \bigr)^{2} \\ &\quad\times{} \bigl(f_{\lambda}(u)+f_{\rho}(u)-2y \bigr)^{2}\,d\rho (u,y) \\ &\leq\bigl(3M+ \Vert f_{\lambda} \Vert _{\infty} \bigr)^{2} \Vert f_{\lambda}-f_{\rho} \Vert _{\rho _{X}}^{2} \\ &\leq B_{\lambda}\mathcal{D}(\lambda). \end{aligned}$$

By Lemma C.1, with confidence $1-\delta/2$, we have

$$\begin{aligned} \frac{1}{m}\sum_{i=1}^{m}g(z_{i})- \int_{Z}g\,d\rho&\leq\frac {4B_{\lambda}\log(2/\delta)}{3m}+\sqrt{\mathcal{D}( \lambda)\times \frac{2B_{\lambda}\log(2/\delta)}{m}} \\ &\leq\frac{\mathcal{D}(\lambda)}{2}+\frac{7B_{\lambda}\log (2/\delta)}{3m} . \end{aligned}$$

This completes the proof of Proposition 3.5. □

We estimate $\mathcal{S}_{1}(\mathbf{z},\eta)$ by using the proposition from [22] below.

### Proposition C.1

*Let* $\mathcal{F}$ *be a class of bounded measurable functions*. *Assume that there are constants* $Q, \tau>0$ *and* $\alpha\in[0,1]$ *such that* $\|f\|_{\infty}\leq Q$ *and* $\mathbb{E}f^{2}\leq\tau\mathbb {E}f^{\alpha}$ *for every* $f\in\mathcal{F}$. *If for some* $a>0$ *and* $0< p<2$,

C.1 $$\begin{aligned} \log\mathcal{N}_{2}(\mathcal{F},\varepsilon)\leq a\varepsilon^{-p},\quad \forall\varepsilon>0, \end{aligned}$$

*then there exists a constant* $c_{p}'$ *depending only on* *p* *such that*, *for any* $t>0$, *with probability at least* $1-e^{-t}$, *we have*

$$\begin{aligned} \mathbb{E}f-\frac{1}{m}\sum_{i=1}^{m}f(z_{i}) \leq&\frac{1}{2}\eta ^{1-\alpha}(\mathbb{E}f)^{\alpha}+c_{p}' \eta+2 \biggl(\frac{\tau t}{m} \biggr)^{\frac{1}{2-\alpha}}+\frac{18Qt}{m}, \end{aligned}$$

*where*

$$\begin{aligned} \eta:=\max \biggl\{ \tau^{\frac{2-p}{4-2\alpha+p\alpha}} \biggl(\frac {a}{m} \biggr)^{\frac{2}{4-2\alpha+p\alpha}}, Q^{\frac{2-p}{2+p}} \biggl(\frac{a}{m} \biggr)^{\frac{2}{2+p}} \biggr\} . \end{aligned}$$

### Proof of Proposition 3.6

Consider the set of functions

$$\begin{aligned} \mathcal{G}_{R}&= \biggl\{ g(u,y)= \int_{X}\Phi \biggl(\frac{x}{\sigma },\frac{u}{\sigma} \biggr) \bigl(\bigl(y-\pi_{M}(f) (u)\bigr)^{2} \\ &\quad {}-\bigl(y-f_{\rho}(u)\bigr)^{2} \bigr)\,d\rho_{X}(x):f \in B_{R} \biggr\} . \end{aligned}$$

By (1.5),

$$\begin{aligned} \bigl\vert g(u,y) \bigr\vert &\leq \int_{X}\Phi \biggl(\frac{x}{\sigma},\frac{u}{\sigma } \biggr) \bigl\vert \bigl(\pi_{M}(f) (u)-f_{\rho}(u) \bigr) \\ &\quad\times{} \bigl(\pi_{M}(f) (u)+f_{\rho}(u)-2y \bigr) \bigr\vert \,d\rho _{X}(x) \\ &\leq8M^{2}. \end{aligned}$$

It follows from the Schwarz inequality that

$$\begin{aligned} \bigl\vert g(u,y) \bigr\vert ^{2}&=\biggl| \int_{X}\Phi \biggl(\frac{x}{\sigma},\frac {u}{\sigma} \biggr) \bigl(\pi_{M}(f) (u)-f_{\rho}(u) \bigr) \\ &\quad\times{} \bigl(\pi_{M}(f) (u)+f_{\rho}(u)-2y \bigr)\,d\rho _{X}(x)\biggr|^{2} \\ &\leq16M^{2} \int_{X}\Phi \biggl(\frac{x}{\sigma},\frac{u}{\sigma } \biggr) \bigl(\pi_{M}(f) (u) \\ &\quad{}-f_{\rho}(u) \bigr)^{2}\,d\rho_{X}(x) \int_{X}\Phi \biggl(\frac {x}{\sigma},\frac{u}{\sigma} \biggr)\,d\rho_{X}(x). \end{aligned}$$

Hence,

$$\begin{aligned} \mathbb{E}\bigl(g^{2}\bigr)\leq16M^{2} \int_{X} \biggl(& \int_{X}\Phi \biggl(\frac{x}{\sigma},\frac{u}{\sigma} \biggr) \bigl(\pi _{M}(f) (u)-f_{\rho}(u) \bigr)^{2}\,d \rho_{X}(u) \biggr)\,d\rho _{X}(x). \end{aligned}$$

It has been proved in [8] that

$$\begin{aligned} & \int_{X}\Phi \biggl(\frac{x}{\sigma},\frac{u}{\sigma} \biggr) \bigl(f(u)-f_{\rho}(u) \bigr)^{2}\,d\rho_{X}(u) \\ &\quad= \int_{Z}\Phi \biggl(\frac{x}{\sigma},\frac{u}{\sigma} \biggr)\bigl[\bigl(f(u)-y\bigr)^{2}-\bigl(f_{\rho}(u)-y \bigr)^{2}\bigr]\,d\rho(u,y), \end{aligned}$$

which implies

$$\begin{aligned} \mathbb{E}\bigl(g^{2}\bigr)&\leq16M^{2} \int_{X} \biggl( \int_{Z}\Phi \biggl(\frac{x}{\sigma},\frac{u}{\sigma} \biggr)\bigl[\bigl(\pi _{M}(f) (u)-y\bigr)^{2} \\ &\quad {}-\bigl(f_{\rho}(u)-y\bigr)^{2}\bigr]\,d \rho(u,y) \biggr)\,d\rho _{X}(x) \\ &=16M^{2} \int_{Z} \biggl( \int_{X}\Phi \biggl(\frac{x}{\sigma},\frac {u}{\sigma} \biggr)\bigl[\bigl(\pi_{M}(f) (u)-y\bigr)^{2} \\ &\quad {}-\bigl(f_{\rho}(u)-y\bigr)^{2}\bigr]\,d \rho_{X}(x) \biggr)\,d\rho (u,y) \\ &=16M^{2}\mathbb{E}(g). \end{aligned}$$

Then, for any $g_{1}$, $g_{2}\in\mathcal{G}_{R}$, we get

$$\begin{aligned} & \bigl\vert g_{1}(u,y)-g_{2}(u,y) \bigr\vert \\ &\quad= \biggl\vert \int_{X}\Phi \biggl(\frac{x}{\sigma},\frac{u}{\sigma} \biggr) \bigl(\bigl(\pi_{M}(f_{1}) (u)-y \bigr)^{2}-\bigl(\pi_{M}(f_{2}) (u)-y \bigr)^{2} \bigr)\,d\rho _{X}(x) \biggr\vert \\ &\quad \leq\biggl| \int_{X}\Phi \biggl(\frac{x}{\sigma},\frac{u}{\sigma } \biggr) \bigl(\pi_{M}(f_{1}) (u)\bigr)-\pi_{M}(f_{2}) (u)) \\ &\qquad\times{}\bigl(\pi_{M}(f_{1}) (u)+ \pi_{M}(f_{2}) (u)-2y\bigr)\,d\rho _{X}(x)\biggr| \\ &\quad \leq4M \bigl\vert f_{1}(u)-f_{2}(u) \bigr\vert , \end{aligned}$$

which implies

$$\begin{aligned} \mathcal{N}_{2}(\mathcal{G}_{R},\varepsilon)\leq\mathcal {N}_{2} \biggl(B_{R},\frac{\varepsilon}{4M} \biggr)=\mathcal {N}_{2} \biggl(B_{1},\frac{\varepsilon}{4MR} \biggr). \end{aligned}$$

Thus from the capacity condition (2.1), we have

$$\begin{aligned} \log\mathcal{N}_{2}(\mathcal{G}_{R},\epsilon)\leq c_{p}(4M)^{p}R^{p}\epsilon^{-p}. \end{aligned}$$

Now we can apply Proposition C.1 to $\mathscr{G}$ with $Q=8M^{2}$, $\alpha=1$, $\tau=16M^{2}$ and $a=c_{p}(4M)^{p}R^{p}$. Thus for any $0<\delta<1$, with confidence $1-\delta/2$, we have

$$\begin{aligned} \mathbb{E}g-\frac{1}{m}\sum_{i=1}^{m}g(z_{i})& \leq\frac{\mathbb {E}g}{2}+\frac{176M^{2}}{m}\log \biggl(\frac{2}{\delta} \biggr)+C_{p,M}R^{\frac{2p}{2+p}}m^{-\frac{2}{2+p}}, \end{aligned}$$

where $C_{p,M}=c_{p}'(4M)^{\frac{4}{2+p}}c_{p}^{\frac{2}{2+p}}$.

Moreover, we take $f=f_{\mathbf{z},\sigma,\eta,x}$ and derive the following bound of $f_{\mathbf{z},\sigma,\eta,x}$ by using the same method as in Lemma 3 of [18] and (1.5):

$$\begin{aligned} \Vert f_{\mathbf{z},\eta} \Vert _{K}\leq\kappa m^{1-\frac{1}{q}} \biggl(\frac {M^{2}}{\eta} \biggr)^{\frac{1}{q}}. \end{aligned}$$

Finally, we complete the proof of Proposition 3.6 by taking $R=R_{\eta}=\kappa m^{1-\frac {1}{q}} (\frac{M^{2}}{\eta} )^{\frac{1}{q}}$. □
